# Advances in the Study of the Ubiquitin-Editing Enzyme A20

**DOI:** 10.3389/fphar.2022.845262

**Published:** 2022-05-03

**Authors:** Wenya Bai, Siying Huo, Junjie Li, Jianlin Shao

**Affiliations:** Department of Anesthesiology, First Affiliated Hospital of Kunming Medical University, Kunming, China

**Keywords:** ubiquitination, ubiquitin-editing enzyme A20, inflammatory response, apoptosis, necroptosis, pyroptosis, autophagy

## Abstract

Ubiquitin modification is a common post-translational protein modification and an important mechanism whereby the body regulates protein levels and functions. As a common enzyme associated with ubiquitin modification, the ubiquitin-editing enzyme A20 may be closely associated with the development of numerous pathological processes through its different structural domains. The aim of this paper is to provide an overview of the following: advances in ubiquitination research, the structure and function of A20, and the relationships between A20 and immune inflammatory response, apoptosis, necroptosis, pyroptosis, and autophagy.

## Introduction

A20 was first reported in 1990, the stimulation of tumor necrosis factor alpha (TNFα) can lead to the upregulation of A20 expression in human umbilical vein endothelial cells; hence, the A20 is also known as TNFα-induced protein 3 (TNFAIP3) (Opipari et al., 1990). Furthermore, A20 has the functions of both deubiquitinase and ubiquitin ligase, so it is called ubiquitin editing enzyme (Wertz et al., 2004). Several studies have confirmed that A20 is a potent anti-inflammatory protein that mediates inflammation in mammals, which is attributed to its ability to suppress Nuclear Factor-kappa B (NF‐κB) signaling in a negative feedback manner (Harhaj and Dixit, 2012). However, recently more and more studies have shown that A20 can also play another function that is independently of NF-κB regulation, such as protect cells from death (Priem et al., 2020). Here we review the role of A20 in regulating the following five essential functions: 1) The immune inflammation response, 2) The apoptosis, 3) The necroptosis, 4) The pyroptosis, 5) The Cell autophagy. We further provide insight into potential mechanisms by which A20 functions in the context of a multi-protein ubiquitin-editing complex.

### Advances in Ubiquitination Research

A ubiquitin molecule is a protein polypeptide consisting of 76 amino acid molecules. Ubiquitination is a common post-translational protein modification (Goldstein et al., 1975). In the presence of three successive enzymes (E1 ubiquitin-activating enzyme, E2 ubiquitin-conjugating enzyme, and E3 ligase) and ATP, the ubiquitin molecule is covalently attached to a lysine residue of the substrate protein through its terminal carboxyl group, then making the formation of monomeric or polyubiquitination modifications (Komander, 2009; Komander and Rape, 2012); ubiquitination modifies the substrate protein. First, E1 utilizes ATP and activates ubiquitin. Then, through its cysteine residue, E1 forms a high-energy thioester bond with the C-terminus of the ubiquitin molecule. This is followed by the transfer of the ubiquitin molecule from E1 to the cysteine residue of E2 to form a thioester intermediate. Finally, E3 selectively recognizes the corresponding E2, ubiquitin, and substrate protein, and catalyzes the formation of an isopeptide bond between ubiquitin and a certain lysine residue on the substrate protein. Notably, there are seven lysine residues on the ubiquitin molecule, all of which can attach to the C-terminus of another ubiquitin molecule, resulting in seven polyubiquitin chains with different linkages (K6, K11, K27, K29, K33, K48, K63). Alternatively, the N-terminal end of the ubiquitin molecule can be linked to the C-terminal end of another ubiquitin molecule by a peptide bond to form a linear ubiquitin chain (which is also called M1-Linked ubiquitin chain), resulting in the polyubiquitination of the substrate protein (Cohen and Strickson, 2017; Haakonsen and Rape, 2019). Different ubiquitination modifications can exert different physiological effects. For example, K48-Linked polyubiquitination modification can make the substrate protein susceptible to recognition and subsequent proteasomal degradation. Studies show that A20-mediated K48-Linked polyubiquitination modification of receptor-interacting kinase 3 (RIPK3) may be related to the switching between mycobacterium tuberculosis-induced apoptosis and necrosis (Fu et al., 2021). In addition, A20 can enhance the K48-Linked polyubiquitination modification of Tumor necrosis factor receptor-associated factor 6 (TRAF6) to suppress autophagy under hypoxic conditions, then inhibiting osteoclastogenesis in human periodontal ligament cells (Yan et al., 2020). A20 can mediate K48-Linked polyubiquitination modification of RNF138 for proteasomal degradation to affect NF-κB activation and lymphomagenesis (Yu et al., 2021); K63-Linked and M1-Linked polyubiquitination modification is involved in serving as a scaffolding network for the formation of signaling complexes. Studies indicated that A20 can downregulate the K63-Linked polyubiquitination modification of TRAF6, which is involved in the anti-neuroinflammatory effects of bavachin (Wang H et al., 2021); In addition, A20 can also play an anti-microbial host defense role through affecting the K63-Linked polyubiquitination modification of different substrate proteins (Liu L. et al., 2020; Liu et al., 2021); Polyubiquitination at K6 is correlated with the development of nonalcoholic steatohepatitis; K29-Linked polyubiquitination is associated with the innate cellular immunity of macrophages (Wang et al., 2020; Gao et al., 2021; Tracz and Bialek, 2021).

The process of polyubiquitination modification is a reversible process; the substrate protein can also undergo deubiquitination by deubiquitinating enzymes (DUBs). Currently, more than 100 DUBs have been identified, which are classified into the following six families based on their structural domains: ubiquitin C-terminal hydrolases, ubiquitin-specific proteases, ovarian tumor domain proteases (OTUs), Josephins, JAB1/MPN/MOV34 (JAMMs) and MCPIP. Of these, the ubiquitin-editing enzyme A20 is by far the most studied DUB of the OTU family. The common feature of DUBs is that they contain specific protein recognition structural domains, such as the zinc finger ubiquitin-specific protease domain (ZnF-UBP), the ubiquitin-interacting motif (UIM) and the ubiquitin-binding structural domain (UBA), which is closely involved in the function of DUBs.

### The Structure and Function of A20

A20 is a member of the OTU family and has a molecular weight of 90 kDa, Subsequent studies have identified two NF-κB binding sites in the promoter of A20, which demonstrating that A20 can be transcriptionally upregulated by Nuclear Factor-kappa B (NF-κB) in response to stimuli (e.g., TNF, IL-1, ROS, virus), providing negative feedback control of NF-κB signaling pathway (Pujari et al., 2013). A20 has an OTU structural domain at the N-terminal end, which can hydrolyze the K63-Linked polyubiquitin chains, to initially regulate signal transduction. Moreover, the C-terminus has seven tandem zinc finger structural domains (ZnF4 and ZnF7 domains are common). The ZnF4 structural domain has an E3 ubiquitin ligase function and can catalyze the formation of K48-Linked polyubiquitin chains, to promote degradation of key signaling molecules; the ZnF7 structural domain, however, readily binds to M1-Linked ubiquitin chains; besides, A20 can also disrupts the interactions between E2 and E3, thus attenuates the activation of specific E3 ligases, then disrupts the ubiquitination cascade (Shembade and Harhaj, 2010; Martens et al., 2020). Owing to its deubiquitinating and ubiquitin ligase activities, A20 is called ubiquitin-editing enzyme A20, which can regulate different different pathological processes in a catalytic and non-catalytic manner.

### A20 and the Immune Inflammatory Response

When an organism receives a harmful external stimulus, such as infection or tissue and organ damage, an inflammatory response occurs. The inflammatory response converts the external stimulus into biological responses through multiple signaling pathways. The NF-κB signaling pathway is one of the main signaling pathways involved in the molecular transduction of inflammatory signals. When stimulated by external signals, such as IL-1β, TNF-α as well as lipopolysaccharide (LPS), IκBα will be degraded and phosphorylated under the stimulation of upstream signaling molecules TGFβ-activated kinase (TAK1) and inhibitor of κBα kinase (IKK) complexes, allowing NF-κB to enter into the nucleus and activate target genes (Hinz et al., 2012). Under normal physiological conditions, A20 is not expressed (or expressed at very low levels) in most cell types, but when NF-κB is activated, NF-κB can transcriptionally activates the expression of A20 *via* the two NF-κB sites in the A20 promoter (Krikos et al., 1992). Several studies have shown that A20 is closely associated with the pathological processes involved in inflammatory and autoimmune diseases, including inflammatory bowel disease (Zhou et al., 2021), rheumatoid arthritis (Malynn and Ma, 2019), chronic inflammatory lung disease (Momtazi et al., 2019), multiple atherosclerotic disease (Perga et al., 2021), lupus nephritis (Sun et al., 2019), and atherosclerosis (Angolano et al., 2021), which is mostly related to that A20 can inhibit the NF-κB signaling pathway in a feedback manner. A20 may inhibit the NF-κB signaling pathway through many mechanisms: 1) During the inflammatory response mediated by IL-1R/Toll-like receptor (TLR4), A20 may inhibit the NF‐κB signaling pathway through the two mechanisms: 1) A20 expression is induced and directly target the E3 ligase TRAF6 by disassembling the K63-Linked polyubiquitin chains of TRAF6 via its OTU domain; 2) A20 disrupt the interactions between TRAF6 and its E2 enzymes Ubc13 and UbcH5 cooperating with the ubiquitin-binding adaptor molecule TAX1BP1 in the first step, then at later times A20 conjugate K48-Linked polyubiquitin chains on Ubc13 and/or UbcH5c via its ZnF4 domain to trigger their proteasomal degradation (Martens and van Loo, 2020). During a TNF-mediated inflammatory response, A20 may hydrolyze the K63-Linked polyubiquitin chain of receptor-interacting kinase 1 (RIPK1) through its OTU structural domain or cause K48-Linked polyubiquitination through the ZnF4 structural domain, thus inhibiting the NF-κB signaling pathway (Shembade and Harhaj, 2012); In addition, A20 can also disrupt the association between E3 ligase complex cellular inhibitor of apoptosis protein 1/2 (cIAP1/2), TRAF2/5 and E2 ubiquitin-conjugating enzyme Ubc13 followed by triggering the ubiquitination and proteasomal degradation of Ubc13 at later time points (Shembade et al., 2010) 3) A20 can also regulate the activation of NF-κB signaling pathway induced by T cell receptor (TCR) and nucleotide oligomerization domain 2 (NOD2) by affecting the ubiquitination level of MALT1 and RIP2, respectively (Hitotsumatsu et al., 2008; Duwel et al., 2009). Furthermore, in the feedback regulation, the ubiquitin modifying functions of A20 can be further enlarged depended on IKKβ-mediated phosphorylation of A20 at Ser381 (Hutti et al., 2007). Taken together, it can be concluded that A20 may inhibit NF-κB signaling pathway through distinct mechanisms in a catalytic manner. Chen et al. found that the downregulation of A20 expression can, through the activation of the NF-κB and mitogen-activated protein kinase (MAPK) signaling pathways, exacerbate the inflammatory response induced by medium-wave ultraviolet radiation (Chen et al., 2020). Studies also found that A20 deficient mice is characterized by the infiltration of CD8^+^ T cells into the CNS, which results in abnormal microglia activation and altered neuronal activity (Mohebiany et al., 2020). In addition, formononetin upregulates A20 *via* G-protein-coupled receptors, which attenuates atopic dermatitis (Yuan et al., 2021). Furthermore, the LINC01140/miR-23/A20 signaling axis alleviates low density lipoprotein (LDL)-induced inflammatory responses in macrophages (He et al., 2020).

**FIGURE 1:**
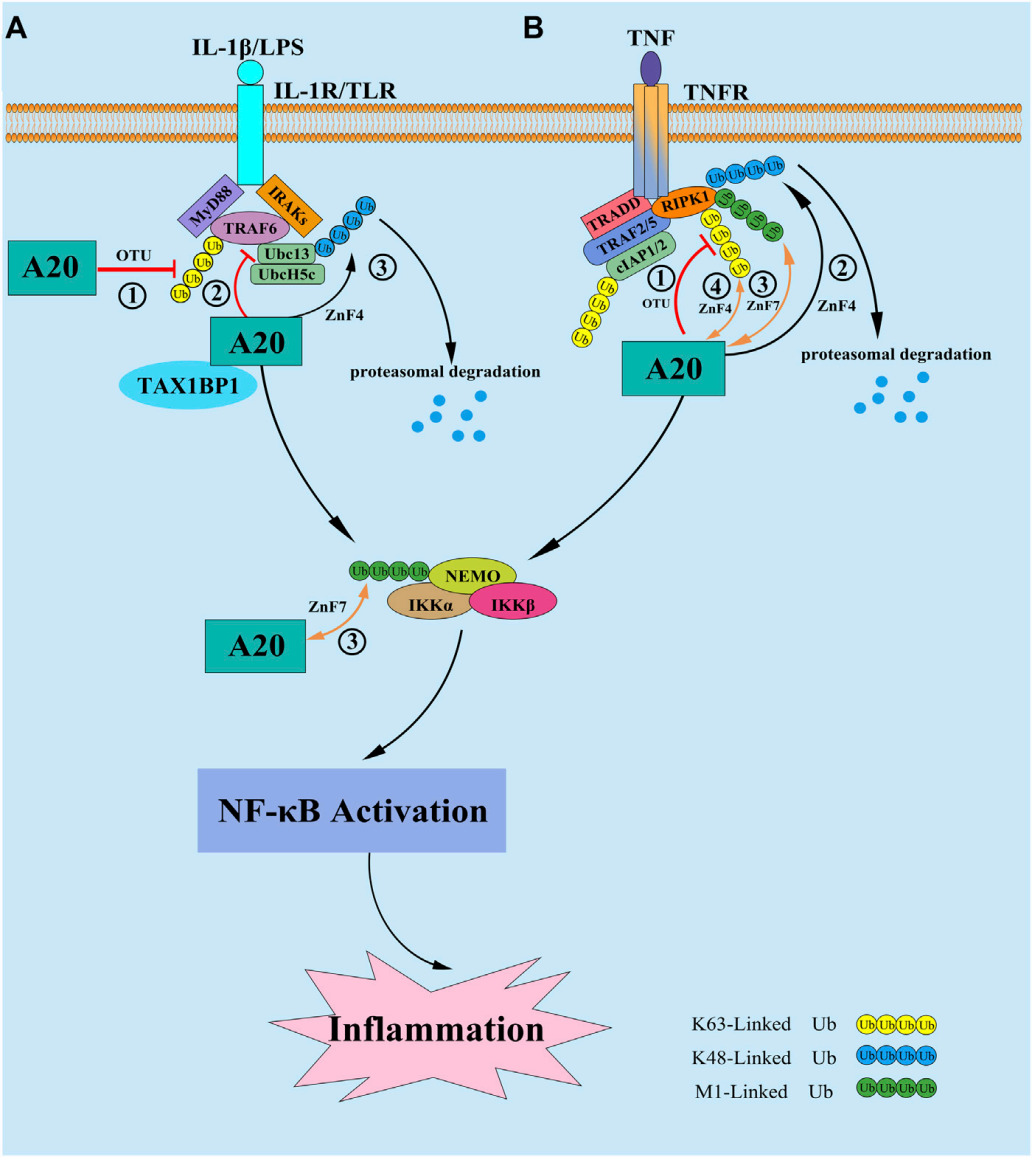
**(A)** In response to IL-1β and/or LPS stimulation, A20 expression is induced and inhibit the NF-κB signaling in a negative feedback loop. ① A20 directly target the E3 ligase TRAF6 by disassembling the K63-Linked polyubiquitin chains of TRAF6 via its OTU domain; ② A20 disrupt the interactions between TRAF6 and its E2 enzymes Ubc13 and UbcH5 cooperating with the ubiquitin-binding adaptor molecule TAX1BP1 in the first step; ③ then at later times A20 conjugate K48-Linked polyubiquitin chains on Ubc13 and/or UbcH5c via its ZnF4 domain to trigger their proteasomal degradation. **(B)** In response to TNF stimulation, A20 is reported to inhibit the NF-κB signaling by the following mechanisms: ① A20 hydrolyze the K63-Linked polyubiquitin chains of RIPK1 *via* its OTU domain to supress the downstream activation of IKK complex, as well as the NF-κB signaling; ② A20 conjugate K48-Linked polyubiquitin chains on RIPK1 *via* its ZnF4 domain to trigger its proteasomal degradation; ③ A20 bind to the M1-Linked polyubiquitin chains of RIPK1 and/or NF-κB essential modulator (NEMO) through its ZnF7 structural domain, to stabilize the M1-Linked polyubiquitin chains and protect them from proteasomal degradation, thus affecting the activation of the IKK complex in a non-catalytic pathway; ④ A20 bind to the K63-Linked polyubiquitin chains of RIPK1 through the ZnF4 domain, which is largely dispensable for restricting NF-κB signaling.

Although A20 is thought to inhibit NF-κB signaling pathway by affecting the ubiquitination levels of multiple target proteins, the physiological role of A20 in regulation of NF-κB was still incompletely clarified. Studies showed that different from A20^−/−^mice, mice bearing a point mutation in either the OTU domain (A20^OTU/OTU^mice) or ZnF4 domain (A20^ZnF4/ZnF4^) do not develop severe inflammatory phenotype (Lu et al., 2013; Wertz et al., 2015), however, the A20^ZnF7/ZnF7^ mice can spontaneously develop arthritis and other autoimmune diseases. These findings indicate that ZnF7 of A20 may play a more prominent role in regulation of NF-κB (Martens et al., 2020; Razani et al., 2020). The mechanism may be related to that A20 can bind to the M1-Linked polyubiquitin chains of RIPK1 and/or NF-κB essential modulator (NEMO) through its ZnF7 structural domain, to stabilize the M1-Linked ubiquitin chain and protect them from deubiquitinase-mediated cleavage, thus affecting the activation of the IKK complex in a non-catalytic manner (Sun, 2020). However, studies have indicated that the inflammatory phenotype of A20^ZnF7/ZnF7^ mice was far less severe than that of A20^−/−^ mice (Lee et al., 2000), and mice carrying mutations both in ZnF7 and ZnF4 causes more serious inflammation and NOD-like receptor p3 (NLRP3) inflammasome activation than that of A20^ZnF7/ZnF7^ mice (Razani et al., 2020), furthermore, Martens and his colleagues have demonstrated that A20 can also bind to the K63-Linked ubiquitin chains of RIPK1 through the ZnF4 domain (Martens et al., 2020), suggesting that ZnF4 and ZnF7 may interact with each other in regulating NF-κB signaling pathway. ZnF4 is largely required for repressing severe inflammatory responses and may play a crucial role in repressing inflammatory response in the absence of a functional ZF7 (Sun, 2020), but both the precise mechanism and whether the E3 ligase activity of ZnF4 is involved in this process need to be further elucidated. Additional studies are urgently needed to further test and determine the functions of ZnF4 and ZnF7 in different physiological status, as well as the role of A20 deubiquitinase catalytic activity, in order to provide a theoretical basis for the treatment of A20-associated autoimmune and inflammatory diseases in the future (Figure 1).

### A20 and Apoptosis

#### A20 Inhibits Apoptosis

**FIGURE 2:**
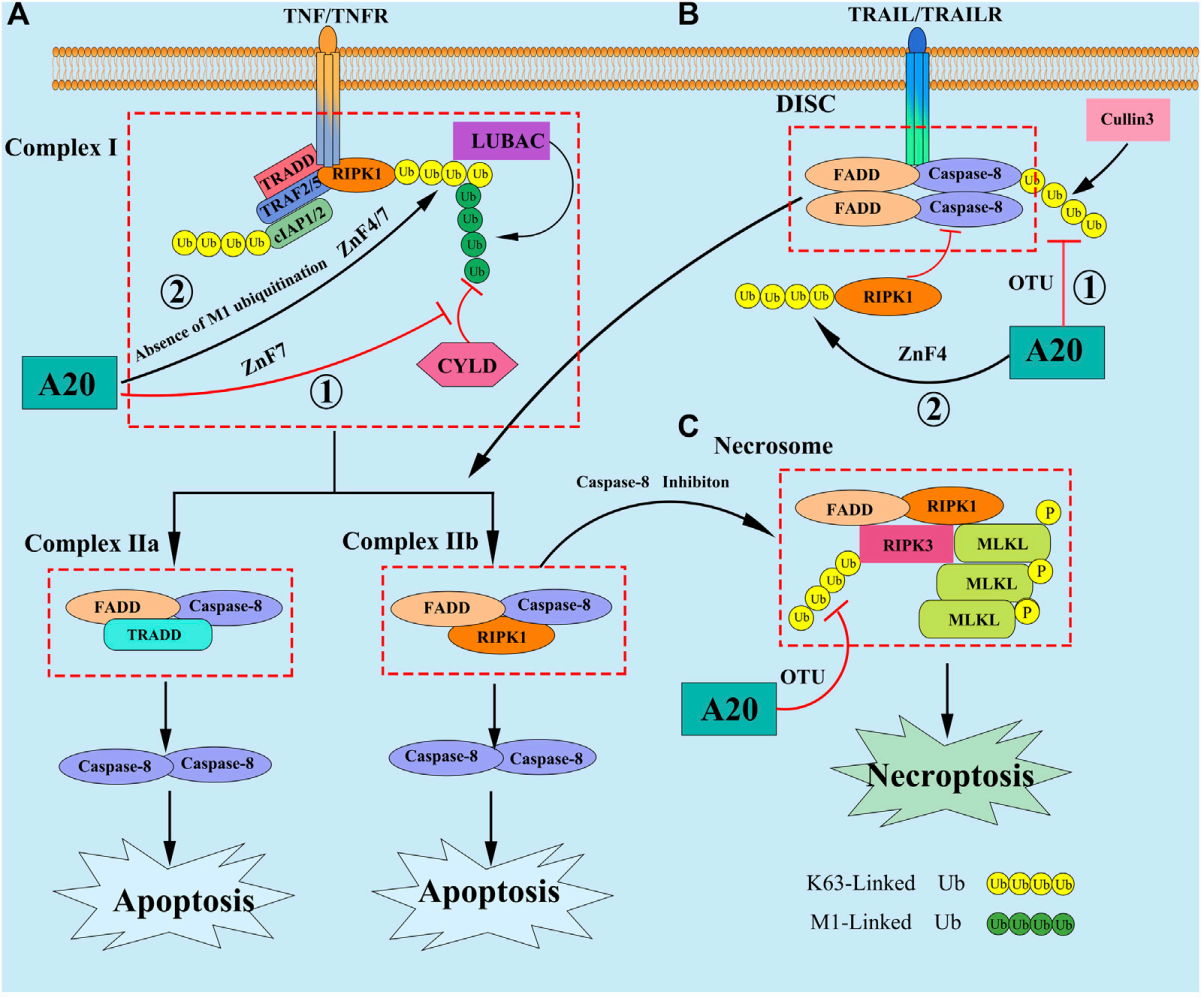
**(A)** In response to TNF stimulation, A20 is reported to inhibit apoptosis by the following mechanisms: ① A20 was shown to be recruited to the membrane-bound complex I, and bound to M1-Linked polyubiquitin chains of RIPK1 *via* its ZnF7 domain, thereby protecting the M1-Linked polyubiquitin chains from CYLD-mediated cleavage and repressing the complex I transitioning to a death-inducing cytosolic complex II; ② In absence of M1-Linked polyubiquitin chains, the ZnF4 and ZnF7 domains of A20 cooperate to bind to other types of ubiquitin chains associated to Complex I, potentially K63-Linked polyubiquitin chains, then protecting cells from apoptosis. **(B)** A20 can also inhibt apoptosis in the TRAIL signaling by the following mechanisms: ① A20 promote the formation of the K63-Linked polyubiquitin chains on RIPK1 through its ZnF4 domain, then RIPK1 being recruited to DISC, and inhibit the dimerization, cleavage of Caspase 8; ② A20 hydrolyze the cullin3-mediated K63-Linked polyubiquitin chains of Caspase-8, then inhibiting caspase-8 activation. **(C)** When Caspase 8 is inhibited, necroptosis can be triggered by DRs, which promote the recruitment of RIPK3 and MLKL to RIPK1 forming Necrosome. Subsequently, RIPK3 can phosphorylate MLKL, resulting in it translocate to the plasma membrane to assemble a pore-forming complex, promoting the release of DAMPs into the environment, thereby inducing necrptosis. A20 restrict the K63-Linked polyubiquitin chains of RIPK3 through its OTU domain, preventing the formation of Necrosome and inhibiting necroptosis.

Apoptosis is a nonlytic form of regulated programmed cell death (PCD), depending on the activation of the death receptor (DRs) extrinsic or mitochondrial intrinsic pathway (Taylor et al., 2008). In response to stimulation by external pro-apoptotic signals, like TNF, TNF-related apoptosis-inducing ligand (TRAIL), Fas, and death receptor 3/4/5 (DR3/4/5), the initiating caspase protein (Caspase-2/8/9/10/11/12) binds to Fas-associated protein with death domain and RIPs, activating the downstream effector caspase proteins (Caspase-3/6/7) and consequently, apoptosis. Studies have found that the upregulation of A20 expression can inhibit apoptosis induced by microglial exosomes (Chen et al., 2019). A20 interacts with RIPK1 through its ZnF7 structural domain to affect caspase-8 protein expression in intestinal epithelial cells, thereby inhibiting their apoptosis (Liu X. et al., 2020). Furthermore, A20 upregulation inhibits intestinal epithelial cell apoptosis in Crohn’s disease (Zhou et al., 2019) by affecting the functions of tumor necrosis factor receptor 1 (TNFR1), TNFR1-associated death domain protein (TRADD) and RIPK1. In a mouse model with hepatic damage, A20 inhibited the apoptosis of hepatocytes and immune inflammation through the NF-κB signaling pathway, playing a role in the chronic rejection of allogeneic liver transplantation (Li et al., 2019; Jia et al., 2021). Although it is fully recognized that A20 is a key regulator that can prevent cells from apoptosis, the mechanism by which it does are still not completely elucidated. To date, the function of A20 in the TNF signaling have been most extensively studied. Upon binding of TNF to TNFR, A20 was shown to be recruited to the membrane-bound complex I, which is consist of RIPK1, TRADD, TRAF2/5, cIAP1/2, etc, and bound to M1-Linked polyubiquitin chains of RIPK1 *via* its ZnF7 domain within minutes, thereby protecting the M1-Linked ubiquitin chains from cylindromatosis (CYLD)-mediated cleavage and repressing the complex I transitioning to a death-inducing cytosolic complex Ⅱ (Ⅱa: FADD/Caspase-8/TRADD; Ⅱb: FADD/Caspase-8/RIPK1) (Wilson et al., 2009; Draber et al., 2015). Recently, another study showed that in M1-Linked polyubiquitin chains proficient cells, the ZnF7 domain may play a dominant role in A20’s prosurvival function, but in absence of M1-Linked polyubiquitin chains, the ZnF4 and ZnF7 domains of A20 cooperate to bind to other types of ubiquitin chains associated to Complex I, potentially K63-Linked chains, then protecting cells from apoptosis by deploying its DUB activity (Priem et al., 2019). Except in the TNF signaling, A20 has been also reported to prevent cells from apoptosis in TRAIL signaling. The studies showed that A20 can promote the formation of the K63-Linked polyubiquitin chains on RIPK1 through its ZnF4 domain, then RIPK1 being recruited to death-inducing signaling complex (DISC), and inhibit the dimerization, cleavage of caspase 8, finally suppress TRAIL-induced apoptosis (Bellail et al., 2012; Guo et al., 2018). In addition, A20 can hydrolysis the cullin3-mediated K63-Linked ubiquitination of caspase-8 on the C-terminal region through its OTU domain, then inhibiting caspase-8 activation (Jin et al., 2009; Lim et al., 2017) (Figure 2). Thus, in summary, A20 appears to have the ability to regulate apoptosis in either a catalytic and/or non-catalytic manner, targeting the function of A20 might therefore be of putative therapeutic value in many diseases.

#### A20 Promotes Apoptosis

Paradoxically, some studies also found that A20 can promote apoptosis in some cell types. Kool et al. reported that A20-deficient Dendritic cells were resistant to apoptosis, caused by upregulation of antiapoptotic proteins Bcl-2 and Bcl-x, which can be transcriptional regulated by NF-κB (Kool et al., 2011). A similar phenomenon was found in A20-deficient B cells and Nasopharyngeal Carcinoma cells (Tavares et al., 2010; Zheng et al., 2017; Fan et al., 2021). Another study suggested that elevated A20 can stabilize the ripoptosome and potentiate its apoptosis-inducing activity, thereby, promote the RIPK1-dependent apoptosis in intestinal epithelial cells (Garcia-Carbonell et al., 2018). Wang et al. also reported that Dot1L transcriptionally activates A20, contributing to RIPK1-dependent apoptosis after cerebral ischemia-reperfusion (Wang J et al., 2021). Recently, a new mechanism related to A20 promoting apoptosis was put forward. The study showed that A20 can repress the expression of its target gene cIAP1/2 through the canonical NF-κB signaling. The changes in cIAP1/2 protein levels promote NIK stabilization and subsequent activation of noncanonical NF-κB signaling. The activated noncanonical NF-κB signaling can further enhance the NIK stabilization in an autocrine manner. Finally, stabilized NIK promotes the formation of the ripoptosome and the execution of cell death (Feoktistova et al., 2020).

Taken together, we assume that A20 play the paradoxically role in apoptosis may be relevant to the different cell types. In addition, the speed at which the target genes respond to the stimulation is worth being paid more attention. But more research will be needed in the future to confirm this hypothesis.

### A20 and Necroptosis

**FIGURE 3:**
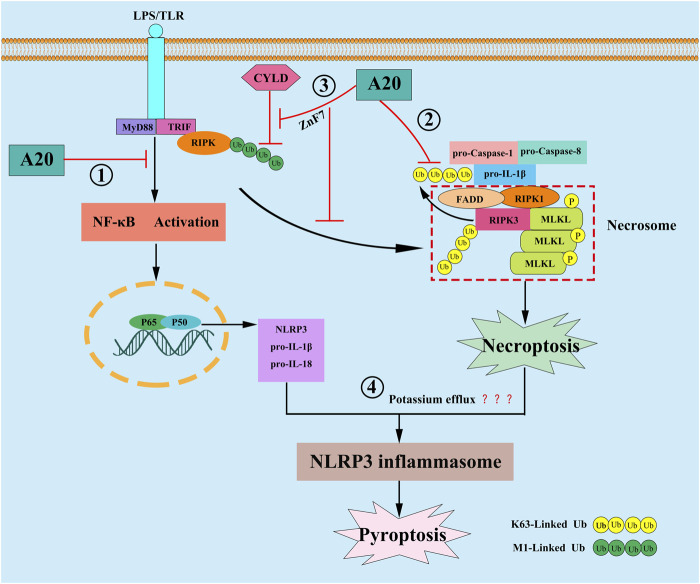
In response to LPS stimulation, A20 is reported to inhibit pyroptosis by the following mechanisms: ① A20 inhibit pyroptosis through inhibiting NF-κB signaling, resulting in the down-regulation of NLRP3 and pro-IL-1β; ② A20 directly interact with the ubiquitylated pro-IL-1β complex, consisting of pro-Caspase-1, pro-Caspase-8, RIPK1 and RIPK3, and then inhibit the formation of RIPK3-dependent K63-Linked polyubiquitin chains of pro-IL-1β, and further repressing the conversion of pro-IL-1β to mature IL-1β; ③ A20 stabilize M1-Linked polyubiquitin chains of RIPK1 by preventing its CYLD-mediated degradation through its ZnF7 domain, then preventing NLRP3 inflammasome activation by inhibiting RIPK1-dependent, RIPK3-MLKL-mediated macrophage necroptosis; ④ A20 may limit necroptosis-induced NLRP3 inflammasome activation by suppressing the potassium efflux.

Necroptosis is a lytic, caspase-independent and calpain dependent form of PCD that can induce inflammatory response. Necroptosis is characterized by the formation of necrosome. Necroptosis shares upstream signaling elements with apoptosis, but the outcomes of apoptosis and necroptosis were significantly distinct (Linkermann and Green, 2014). Necroptosis can be triggered by DRs, which promote the recruitment of RIPK3 and mixed lineage kinase domain-like protein (MLKL) to RIPK1 forming RIPK1-RIPK3-MLKL signaling complex, which is also called necrosome. Subsequently, RIPK3 can phosphorylate MLKL, and phosphorylated MLKL translocate to the plasma membrane to assemble a pore-forming complex, promoting the release of danger-associated molecular patterns (DAMPs) into the environment, thereby inducing necrptosis (Jin et al., 2009). Of note, necroptosis appears to have evolved as a backup suicide mechanism, when caspase-8 is inhibited (Kaiser et al., 2011; Zhang et al., 2011). Studies show that melatonin can inhibit RIPK3-mediated microglial necroptosis by up-regulating the A20 expression after ICH (Lu et al., 2019). Studies also demonstrated that A20 was indispensable for regulating and controlling necroptosis after traumatic brain injury. They also found that A20 - silencing lead to aggressive necroptosis and attenuate the anti-necroptotic effects of necrostatin-1 and melatonin (Bao et al., 2019). Lysosomal degradation of A20 has been reported to be related to the necroptosis of endothelial cells in Alzheimer’s disease (Zou et al., 2020). Resveratrol can relieve chlorothalonil-induced necroptosis through up-regulation of A20 in fish kidney cells (Li et al., 2020). Taken together, these studies can be concluded that A20 can ameliorating different kinds of disease, not only by suppressing apoptosis, but also necroptosis. The underlying mechanism by which A20 regulating necroptosis may be related to that A20 can inactive RIPK3 by regulating the K63-Linked polyubiquitination modification of RIPK3, then suppressing necroptosis (Zhou et al., 2021). Onizawa et al. also found that A20 deficient T cells and fibroblasts are susceptible to caspase independent and RIPK3 dependent necroptosis, which is related to that A20 can restrict the K63-Linked polyubiquitination modification of RIPK3 through its deubiquitinating motif, preventing the formation of RIPK1-RIPK3 complexes and inhibiting necroptosis (Onizawa et al., 2015). Moreover, another study demonstrated that in the LPS/TLR signaling, A20 can prevent NLRP3 inflammasome activation by inhibiting RIPK1 kinase-dependent, RIPK3 - MLKL-mediated macrophage necroptosis, through its ZnF7 domain, and this process may be closely related to that A20 can stabilize M1-Linked ubiquition chains of RIPK1 by preventing its degradation by CYLD, which is also relevant for its function in repressing pyroptosis (Polykratis et al., 2019). Besides, it has been reported that A20 can inhibit necroptosis by affecting the activity of RIPK1 (Iorga et al., 2021; Wang Y et al., 2021) (Figures 2, 3). Taken together, there may be overlapping function of A20 in regulating necroptosis and apoptosis, as well as pyroptosis (see in the next section), which deserve being paid more attention to. The overlapping function of A20 in different pathways may have important implications for the treatment of chronic inflammatory diseases in the future.

**FIGURE 4:**
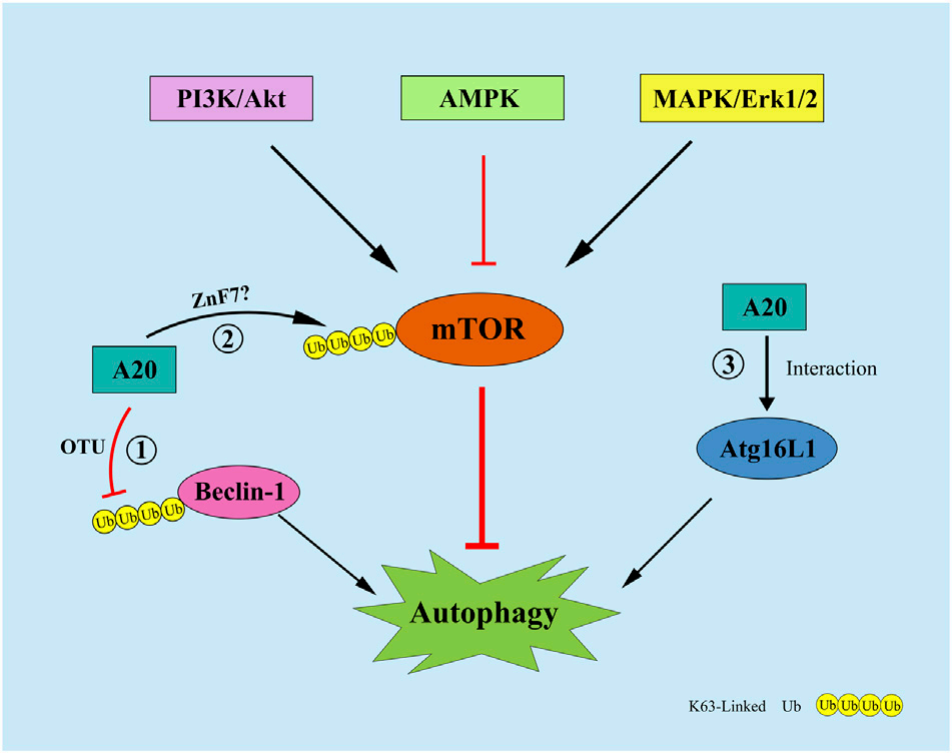
A20 can regulate autophagy by the following mechanisms: ① A20 remove the K63-Linked polyubiquitin chains of Beclin1 through its OTU domain; ② A20 affect the ubiquitination level of mTOR through its ZnF7 domain; ③ A20 interact with ATG16L1 to regulate autophagy.

### A20 and Pyroptosis

Pyroptosis is another form of PCD that is dependent on NLRP3 inflammasomes. The process is accompanied by the release of numerous inflammatory cytokines and is therefore also known as “inflammatory necrosis” (Lamkanfi and Dixit, 2014). NLRP3 expression is upregulated by the upstream transcription factor, NF-κB. Subsequently, ASC and pro-caspase-1 proteins are recruited and assembled to form NLRP3 inflammasomes. This is followed by the activation of caspase-1, which in turn shears gasdermin D (GSDMD) to active GSDMD-N; the latter can induce pore formation and cause leakage of cellular contents such as IL-1β and IL-18 as well as the massive efflux of potassium ions, leading to cell swelling and lysis (Cao et al., 2021). In cerebral hemorrhage, the upregulation of A20 expression can inhibit RIPK3-mediated programmed cell death (Lu et al., 2019). In diseases such as osteoarthritis (Vande et al., 2014), endotoxic shock (Xue et al., 2019), diabetic nephropathy (Ding et al., 2021), Parkinson’s disease (Mouton-Liger et al., 2018), and neuroinflammation (Voet et al., 2018). A20 can inhibit pyroptosis by suppressing the activation of NLRP3 inflammasomes. Studies have found that A20 can inhibit pyroptosis through inhibiting NF-κB signaling, resulting in the down-regulation of NLRP3 and pro-IL-1β (Cornut et al., 2020; Lopez-Castejon, 2020). Nevertheless, some studies showed that blocking NF-κB cannot reverse the arthritic phenotype in A20-deficient mouse, suggesting that there might be another mechanism that concerning A20 regulation pyroptosis. The studies proposed that after LPS engagement, A20 can directly interact with the ubiquitylated pro-IL-1β complex, consisting of pro-caspase-1, pro-caspase-8, RIPK1 and RIPK3, which formed prior to assembly of the NLRP3 inflammasome, and then inhibit the formation of RIPK3-dependent K63-Linked ubiquitination chains of pro-IL-1β, and further repressing the conversion of pro-IL-1β to mature IL-1β (Vande et al., 2014; Duong et al., 2015). Moreover, another study demonstrated that A20 can prevent NLRP3 inflammasome activation by inhibiting RIPK1 kinase-dependent, RIPK3-MLKL-mediated macrophage necroptosis through its ZnF7 domain, and this process may be closely related to that A20 can stabilize M1-Linked ubiquition chains of RIPK1 by preventing its CYLD-mediated degradation by CYLD (Polykratis et al., 2019). Martens et al. also found that A20^ZnF7/ZnF7^ BMDMs showed significantly enhanced NLRP3 inflammasome mediated caspase-1 activation, pyroptosis and IL-1β and IL-18, they illustrated that the ZnF7 domain of A20 is very for preserving TNFR1 complex integrity, and for preventing cell death, inflammasome activation and inflammation. which reenforce the idea that A20 can utilize its ZnF7 domain to restrict the activation of NLRP3 inflammasome in macrophage (Vande et al., 2014; Martens et al., 2020). However, Razani et al. reported the exact opposite result. They found that despite increased LPS induced responses, A20^ZnF7/ZnF7^ macrophages failed to secrete IL-1β after stimulation with LPS alone, furthermore arthritis in A20^ZnF7/ZnF7^mice is not abrogated by NLRP3 inflammasome deficiency (Razani et al., 2020). We think that these contradictory results may be related to the following reasons: First, Razani et al. only utilized LPS stimulated the A20^ZnF7/ZnF7^ macrophages, but Martens et al. utilized LPS plus ATP, since extracellular ATP is indispensable in activating the NLRP3 inflammasome in the second signal, perhaps the use of ATP or not may make a difference, and Duong et al. also reported that LPS alone cannot stimulate A20^−/−^BMDM cells secrete additional IL-1β, in contrast, addition of ATP to these cells induced robust IL-1β secretion (Duong et al., 2015); Second, the ZnF4 domain may also play a synergistic role in the regulation of NLRP3 inflammasome activation, because A20^ZF4ZF7/ZF4ZF7^ macrophages displayed spontaneous NLRP3 inflammasome activity. But these are just our assumptions and have not been confirmed by more detailed experiments. Taken together, the link between ZnF7 domain of A20 and NLRP3 is controversial, more studies will be need to clarify the function of ZnF7, especially the coordinated function of ZnF7 and ZnF4. Studies also showed that RIPK3-MLKL necroptotic signaling can activate the NLRP3 inflammasome in a cell-intrinsic manner. They also demonstrated that oligomerization and association of MLKL with cellular membranes, and a reduction in intracellular potassium concentration may be linked to MLKL-induced necroptosis with NLRP3 activation (Munoz-Planillo et al., 2013; Conos et al., 2017), so we think that A20 may limits necroptosis-induced NLRP3 inflammasome activation by suppressing the potassium efflux, but more research will be required to fully confirm this hypothesis. In summary, A20 can regulate pyroptosis not only by inhibiting NF-κB-dependent NLRP3 and pro-IL-1β upregulation, but also by repressing the formation of RIPK3-dependent K63-Linked polyubiquitin chains of pro-IL-1β, and necroptosis-inducing NLRP3 inflammasome activation.

### A20 and Autophagy

Autophagy is a cathepsin-dependent, and highly conserved process for the degradation of intracellular waste (Dikic and Elazar, 2018). Autophagy is divided into Atg5-Atg12-mediated and Beclin1-mediated pathways. The formation of autophagosomes possessing double-membrane vesicles is the most important mechanism for the occurrence of autophagy. Moreover, Beclin1 expression is positively correlated with autophagy levels. Studies have shown that A20 can inhibit TLR4-induced autophagy through its OTU structural domain by removing the K63-Linked polyubiquitin chain from the lysine residue 117 of Beclin1 (Shi and Kehrl, 2010). Vetters et al. showed that A20 can, through its ZnF7 structural domain, affect the ubiquitination level of mTOR and maintain the balance in the quantity of NK and CD4^+^ T cells *in vivo* (Matsuzawa et al., 2015; Vetters et al., 2019). Furthermore, A20 can maintain the stability of the intestinal epithelial barrier by interacting with ATG16L1, a key protein of autophagy (Slowicka et al., 2019). Under hypoxic conditions, A20 inhibits autophagy and thus osteoclastogenesis by affecting the level of TRAF6 ubiquitination (Yan et al., 2020). Moreover, in human ankylosing spondylitis, A20 induces early autophagy by stabilizing mTOR-interacting protein and attenuates the symptoms of the disease (Zhai et al., 2018). There are few studies on the regulation of autophagy by A20, more studies are needed to further determine how A20 affects the ubiquitination levels of mTOR and Beclin1. Moreover, the experiments that relevant to the overlapping function of A20 in regulating autophagy and inflammation, apoptosis, necroptosis, pyroptosis will be more more valuable.

## Conclusion and Perspectives

A20 was initially thought to be a major negative regulator of the NF-κB signaling pathway, and studies on A20 focused on its inhibitory effects on inflammatory responses and autoimmune diseases. In recent years, several studies have confirmed that A20 can not only regulate inflammatory responses, but also play a important in regulating cell death, like apoptosis, necroptosis, pyroptosis, autophagy. Among other mechanisms, it can affect the NF-κB signaling pathway in both catalytic and non-catalytic pathways, hydrolyze the polyubiquitin chain of caspase proteins, inhibit the expression of NLRP3 inflammasomes, and affect the ubiquitination levels of pro-IL-1β, mTOR, Beclin-1, and TRAF6 in both catalytic and noncatalytic pathways. However, it is worth noting that A20 may have different modes of action in different cell types. Therefore, more studies are needed to confirm the specificity of A20 sites of action, which could provide a theoretical basis for presenting A20 as a new target for disease therapy in the future.

## References

[B1] Angolano C. Kaczmarek E. Essayagh S. Daniel S. Choi L. Y. Tung B. (2021). A20/TNFAIP3 Increases ENOS Expression in an ERK5/KLF2-dependent Manner to Support Endothelial Cell Health in the Face of Inflammation. Front. Cardiovasc. Med. 8, 651230. 10.3389/fcvm.2021.651230 34026871 PMC8138474

[B2] Bao Z. Fan L. Zhao L. Xu X. Liu Y. Chao H. (2019). Silencing of A20 Aggravates Neuronal Death and Inflammation after Traumatic Brain Injury: A Potential Trigger of Necroptosis. Front. Mol. Neurosci. 12, 222. 10.3389/fnmol.2019.00222 31607859 PMC6761256

[B3] Bellail A. C. Olson J. J. Yang X. Chen Z. J. Hao C. (2012). A20 Ubiquitin Ligase-Mediated Polyubiquitination of RIP1 Inhibits Caspase-8 Cleavage and TRAIL-Induced Apoptosis in Glioblastoma. Cancer Discov. 2 (2), 140–155. 10.1158/2159-8290.CD-11-0172 22585859 PMC3354650

[B4] Cao X. Wang Y. Gao L. (2021). CHRFAM7A Overexpression Attenuates Cerebral Ischemia-Reperfusion Injury via Inhibiting Microglia Pyroptosis Mediated by the NLRP3/Caspase-1 Pathway. Inflammation 44 (3), 1023–1034. 10.1007/s10753-020-01398-4 33405023

[B5] Chen X. Qian B. Kong X. Hao J. Ye Y. Yang K. (2019). A20 Protects Neuronal Apoptosis Stimulated by Lipopolysaccharide-Induced Microglial Exosomes. Neurosci. Lett. 712, 134480. 10.1016/j.neulet.2019.134480 31493550

[B6] Chen Y. Wu W. Li X. Guo Y. Lu F. Man M. (2020). A20 Down-Regulation Promotes UVB-Induced Inflammation via Activation of the NF-Κb Pathway. J. Dermatol. Sci. 98 (1), 61–64. 10.1016/j.jdermsci.2020.02.007 32169275

[B7] Cohen P. Strickson S. (2017). The Role of Hybrid Ubiquitin Chains in the MyD88 and Other Innate Immune Signalling Pathways. Cell Death Differ. 24 (7), 1153–1159. 10.1038/cdd.2017.17 28475177 PMC5520163

[B8] Conos S. A. Chen K. W. De Nardo D. Hara H. Whitehead L. Núñez G. (2017). Active MLKL Triggers the NLRP3 Inflammasome in a Cell-Intrinsic Manner. Proc. Natl. Acad. Sci. U S A. 114 (6), E961–E969. 10.1073/pnas.1613305114 28096356 PMC5307433

[B9] Cornut M. Bourdonnay E. Henry T. (2020). Transcriptional Regulation of Inflammasomes. Int. J. Mol. Sci. 21 (21), 8087. 10.3390/ijms21218087 PMC7663688 33138274

[B10] Dikic I. Elazar Z. (2018). Mechanism and Medical Implications of Mammalian Autophagy. Nat. Rev. Mol. Cel. Biol. 19 (6), 349–364. 10.1038/s41580-018-0003-4 29618831

[B11] Ding X. Jing N. Shen A. Guo F. Song Y. Pan M. (2021). MiR-21-5p in Macrophage-Derived Extracellular Vesicles Affects Podocyte Pyroptosis in Diabetic Nephropathy by Regulating A20. J. Endocrinol. Invest. 44 (6), 1175–1184. 10.1007/s40618-020-01401-7 32930981

[B12] Draber P. Kupka S. Reichert M. Draberova H. Lafont E. de Miguel D. (2015). LUBAC-recruited CYLD and A20 Regulate Gene Activation and Cell Death by Exerting Opposing Effects on Linear Ubiquitin in Signaling Complexes. Cell Rep. 13 (10), 2258–2272. 10.1016/j.celrep.2015.11.009 26670046 PMC4688036

[B13] Duong B. H. Onizawa M. Oses-Prieto J. A. Advincula R. Burlingame A. Malynn B. A. (2015). A20 Restricts Ubiquitination of Pro-interleukin-1β Protein Complexes and Suppresses NLRP3 Inflammasome Activity. Immunity 42 (1), 55–67. 10.1016/j.immuni.2014.12.031 25607459 PMC4302274

[B14] Düwel M. Welteke V. Oeckinghaus A. Baens M. Kloo B. Ferch U. (2009). A20 Negatively Regulates T Cell Receptor Signaling to NF-kappaB by Cleaving Malt1 Ubiquitin Chains. J. Immunol. 182 (12), 7718–7728. 10.4049/jimmunol.0803313 19494296

[B15] Fan J. Iwata S. Tanaka Y. Kitanaga Y. Ishii A. Maiko H. (2021). Kdm5a Promotes B Cell Activation in Systemic Lupus Erythematosus via Downregulation of A20 by Histone Modification. Pathol. Res. Pract. [Online ahead of print], 153653. 10.1016/j.prp.2021.153653 34763954

[B16] Feoktistova M. Makarov R. Brenji S. Schneider A. T. Hooiveld G. J. Luedde T. (2020). A20 Promotes Ripoptosome Formation and TNF-Induced Apoptosis via cIAPs Regulation and NIK Stabilization in Keratinocytes. Cells 9 (2). 10.3390/cells9020351 PMC7072579 32028675

[B17] Fu B. Lin X. Tan S. Zhang R. Xue W. Zhang H. (2021). MiR-342 Controls *Mycobacterium tuberculosis* Susceptibility by Modulating Inflammation and Cell Death. EMBO Rep. 22 (9), e52252. 10.15252/embr.202052252 34288348 PMC8419689

[B18] Gao P. Ma X. Yuan M. Yi Y. Liu G. Wen M. (2021). E3 Ligase Nedd4l Promotes Antiviral Innate Immunity by Catalyzing K29-Linked Cysteine Ubiquitination of TRAF3. Nat. Commun. 12 (1), 1194. 10.1038/s41467-021-21456-1 33608556 PMC7895832

[B19] Garcia-Carbonell R. Wong J. Kim J. Y. Close L. A. Boland B. S. Wong T. L. (2018). Elevated A20 Promotes TNF-Induced and RIPK1-Dependent Intestinal Epithelial Cell Death. Proc. Natl. Acad. Sci. U S A. 115 (39), E9192–E9200. 10.1073/pnas.1810584115 30209212 PMC6166836

[B20] Goldstein G. Scheid M. Hammerling U. Schlesinger D. H. Niall H. D. Boyse E. A. (1975). Isolation of a Polypeptide that Has Lymphocyte-Differentiating Properties and Is Probably Represented Universally in Living Cells. Proc. Natl. Acad. Sci. U S A. 72 (1), 11–15. 10.1073/pnas.72.1.11 1078892 PMC432229

[B21] Guo T. Zhang Y. Qu X. Che X. Li C. Fan Y. (2018). miR-200a Enhances TRAIL-Induced Apoptosis in Gastric Cancer Cells by Targeting A20. Cell Biol. Int. 42 (5), 506–514. 10.1002/cbin.10924 29274253

[B22] Haakonsen D. L. Rape M. (2019). Branching Out: Improved Signaling by Heterotypic Ubiquitin Chains. Trends Cel. Biol. 29 (9), 704–716. 10.1016/j.tcb.2019.06.003 31300189

[B23] Harhaj E. W. Dixit V. M. (2012). Regulation of NF-Κb by Deubiquitinases. Immunol. Rev. 246 (1), 107–124. 10.1111/j.1600-065X.2012.01100.x 22435550 PMC3540820

[B24] He L. Zhao X. He L. (2020). LINC01140 Alleviates the Oxidized Low-Density Lipoprotein-Induced Inflammatory Response in Macrophages via Suppressing miR-23b. Inflammation 43 (1), 66–73. 10.1007/s10753-019-01094-y 31748847

[B25] Hinz M. Arslan S. Ç. Scheidereit C. (2012). It Takes Two to Tango: IκBs, the Multifunctional Partners of NF-Κb. Immunol. Rev. 246 (1), 59–76. 10.1111/j.1600-065X.2012.01102.x 22435547

[B26] Hitotsumatsu O. Ahmad R. C. Tavares R. Wang M. Philpott D. Turer E. E. (2008). The Ubiquitin-Editing Enzyme A20 Restricts Nucleotide-Binding Oligomerization Domain Containing 2-Triggered Signals. Immunity 28 (3), 381–390. 10.1016/j.immuni.2008.02.002 18342009 PMC3606373

[B27] Hutti J. E. Turk B. E. Asara J. M. Ma A. Cantley L. C. Abbott D. W. (2007). IkappaB Kinase Beta Phosphorylates the K63 Deubiquitinase A20 to Cause Feedback Inhibition of the NF-kappaB Pathway. Mol. Cel. Biol. 27 (21), 7451–7461. 10.1128/MCB.01101-07 PMC2169042 17709380

[B28] Iorga A. Donovan K. Shojaie L. Johnson H. Kwok J. Suda J. (2021). Interaction of RIPK1 and A20 Modulates MAPK Signaling in Murine Acetaminophen Toxicity. J. Biol. Chem. 296, 100300. 10.1016/j.jbc.2021.100300 33460648 PMC7948960

[B29] Jia F. Deng F. Xu P. Li S. Wang X. Hu P. (2021). NOD1 Agonist Protects Against Lipopolysaccharide and D-Galactosamine-Induced Fatal Hepatitis Through the Upregulation of A20 Expression in Hepatocytes. Front. Immunol. 12, 603192. 10.3389/fimmu.2021.603192 33746949 PMC7969647

[B30] Jin Z. Li Y. Pitti R. Lawrence D. Pham V. C. Lill J. R. (2009). Cullin3-Based Polyubiquitination and P62-Dependent Aggregation of Caspase-8 Mediate Extrinsic Apoptosis Signaling. Cell 137 (4), 721–735. 10.1016/j.cell.2009.03.015 19427028

[B31] Kaiser W. J. Upton J. W. Long A. B. Livingston-Rosanoff D. Daley-Bauer L. P. Hakem R. (2011). RIP3 Mediates the Embryonic Lethality of Caspase-8-Deficient Mice. Nature 471 (7338), 368–372. 10.1038/nature09857 21368762 PMC3060292

[B32] Komander D. Rape M. (2012). The Ubiquitin Code. Annu. Rev. Biochem. 81, 203–229. 10.1146/annurev-biochem-060310-170328 22524316

[B33] Komander D. (2009). The Emerging Complexity of Protein Ubiquitination. Biochem. Soc. Trans. 37 (Pt 5), 937–953. 10.1042/BST0370937 19754430

[B34] Kool M. van Loo G. Waelput W. De Prijck S. Muskens F. Sze M. (2011). The Ubiquitin-Editing Protein A20 Prevents Dendritic Cell Activation, Recognition of Apoptotic Cells, and Systemic Autoimmunity. Immunity 35 (1), 82–96. 10.1016/j.immuni.2011.05.013 21723156

[B35] Krikos A. Laherty C. D. Dixit V. M. (1992). Transcriptional Activation of the Tumor Necrosis Factor Alpha-Inducible Zinc finger Protein, A20, Is Mediated by Kappa B Elements. J. Biol. Chem. 267 (25), 17971–17976. 10.1016/s0021-9258(19)37138-8 1381359

[B36] Lamkanfi M. Dixit V. M. (2014). Mechanisms and Functions of Inflammasomes. Cell 157 (5), 1013–1022. 10.1016/j.cell.2014.04.007 24855941

[B37] Lee E. G. Boone D. L. Chai S. Libby S. L. Chien M. Lodolce J. P. (2000). Failure to Regulate TNF-Induced NF-kappaB and Cell Death Responses in A20-Deficient Mice. Science 289 (5488), 2350–2354. 10.1126/science.289.5488.2350 11009421 PMC3582399

[B38] Li K. Z. Liao Z. Y. Li Y. X. Ming Z. Y. Zhong J. H. Wu G. B. (2019). A20 Rescues Hepatocytes From Apoptosis Through the NF-Κb Signaling Pathway in Rats with Acute Liver Failure. Biosci. Rep. 39 (1). 10.1042/BSR20180316 PMC6328859 30446523

[B39] Li X. Yao Y. Wang S. Xu S. (2020). Resveratrol Relieves Chlorothalonil-Induced Apoptosis and Necroptosis Through miR-15a/Bcl2-A20 Axis in Fish Kidney Cells. Fish. Shellfish Immunol. 107 (Pt B), 427–434. 10.1016/j.fsi.2020.11.007 33186708

[B40] Lim M. C. C. Maubach G. Sokolova O. Feige M. H. Diezko R. Buchbinder J. (2017). Pathogen-Induced Ubiquitin-Editing Enzyme A20 Bifunctionally Shuts off NF-Κb and Caspase-8-Dependent Apoptotic Cell Death. Cel. Death Differ. 24 (9), 1621–1631. 10.1038/cdd.2017.89 PMC5563994 28574503

[B41] Linkermann A. Green D. R. (2014). Necroptosis. N. Engl. J. Med. 370 (5), 455–465. 10.1056/NEJMra1310050 24476434 PMC4035222

[B42] Liu L. Wei Z. Fang R. Li X. Li W. (2020a). Giardia Duodenalis Induces Extrinsic Pathway of Apoptosis in Intestinal Epithelial Cells Through Activation of TNFR1 and K63 De-Ubiquitination of RIP1 *In Vitro* . Microb. Pathog. 149, 104315. 10.1016/j.micpath.2020.104315 32525021

[B43] Liu L. Yang Y. Fang R. Zhu W. Wu J. Li X. (2021). Giardia Duodenalis and its Secreted PPIB Trigger Inflammasome Activation and Pyroptosis in Macrophages Through TLR4-Induced ROS Signaling and A20-Mediated NLRP3 Deubiquitination. Cells 10 (12), 3425. 10.3390/cells10123425 34943932 PMC8700504

[B44] Liu X. Mao Y. Kang Y. He L. Zhu B. Zhang W. (2020b). MicroRNA-127 Promotes Anti-Microbial Host Defense Through Restricting A20-Mediated De-Ubiquitination of STAT3. iScience 23 (1), 100763. 10.1016/j.isci.2019.100763 31958753 PMC6992901

[B45] Lopez-Castejon G. (2020). Control of the Inflammasome by the Ubiquitin System. FEBS J. 287 (1), 11–26. 10.1111/febs.15118 31679183 PMC7138099

[B46] Lu J. Sun Z. Fang Y. Zheng J. Xu S. Xu W. (2019). Melatonin Suppresses Microglial Necroptosis by Regulating Deubiquitinating Enzyme A20 After Intracerebral Hemorrhage. Front. Immunol. 10, 1360. 10.3389/fimmu.2019.01360 31258534 PMC6587666

[B47] Lu T. T. Onizawa M. Hammer G. E. Turer E. E. Yin Q. Damko E. (2013). Dimerization and Ubiquitin Mediated Recruitment of A20, a Complex Deubiquitinating Enzyme. Immunity 38 (5), 896–905. 10.1016/j.immuni.2013.03.008 23602765 PMC3665706

[B48] Malynn B. A. Ma A. (2019). A20: A Multifunctional Tool for Regulating Immunity and Preventing Disease. Cell. Immunol. 340, 103914. 10.1016/j.cellimm.2019.04.002 31030956 PMC6584049

[B49] Martens A. Priem D. Hoste E. Vetters J. Rennen S. Catrysse L. (2020). Two Distinct Ubiquitin-Binding Motifs in A20 Mediate its Anti-Inflammatory and Cell-Protective Activities. Nat. Immunol. 21 (4), 381–387. 10.1038/s41590-020-0621-9 32205881

[B50] Martens A. van Loo G. (2020). A20 at the Crossroads of Cell Death, Inflammation, and Autoimmunity. Cold Spring Harb. Perspect. Biol. 12 (1). 10.1101/cshperspect.a036418 PMC6942121 31427375

[B51] Matsuzawa Y. Oshima S. Takahara M. Maeyashiki C. Nemoto Y. Kobayashi M. (2015). TNFAIP3 Promotes Survival of CD4 T Cells by Restricting MTOR and Promoting Autophagy. Autophagy 11 (7), 1052–1062. 10.1080/15548627.2015.1055439 26043155 PMC4590588

[B52] Mohebiany A. N. Ramphal N. S. Karram K. Di Liberto G. Novkovic T. Klein M. (2020). Microglial A20 Protects the Brain from CD8 T-Cell-Mediated Immunopathology. Cel. Rep. 30 (5), 1585–e6. 10.1016/j.celrep.2019.12.097 32023471

[B53] Momtazi G. Lambrecht B. N. Naranjo J. R. Schock B. C. (2019). Regulators of A20 (TNFAIP3): New Drug-Able Targets in Inflammation. Am. J. Physiol. Lung Cel. Mol. Physiol. 316 (3), L456–L469. 10.1152/ajplung.00335.2018 30543305

[B54] Mouton-Liger F. Rosazza T. Sepulveda-Diaz J. Ieang A. Hassoun S. M. Claire E. (2018). Parkin Deficiency Modulates NLRP3 Inflammasome Activation by Attenuating an A20-Dependent Negative Feedback Loop. Glia 66 (8), 1736–1751. 10.1002/glia.23337 29665074 PMC6190839

[B55] Muñoz-Planillo R. Kuffa P. Martínez-Colón G. Smith B. L. Rajendiran T. M. Núñez G. (2013). K⁺ Efflux Is the Common Trigger of NLRP3 Inflammasome Activation by Bacterial Toxins and Particulate Matter. Immunity 38 (6), 1142–1153. 10.1016/j.immuni.2013.05.016 23809161 PMC3730833

[B56] Onizawa M. Oshima S. Schulze-Topphoff U. Oses-Prieto J. A. Lu T. Tavares R. (2015). Erratum: The Ubiquitin-Modifying Enzyme A20 Restricts Ubiquitination of the Kinase RIPK3 and Protects Cells From Necroptosis. Nat. Immunol. 16 (6), 785–627. 10.1038/ni.317210.1038/ni0715-785c PMC4881846 26086145

[B57] Opipari A. W. Boguski M. S. Dixit V. M. (1990). The A20 cDNA Induced by Tumor Necrosis Factor Alpha Encodes a Novel Type of Zinc Finger Protein. J. Biol. Chem. 265 (25), 14705–14708. 10.1016/s0021-9258(18)77165-2 2118515

[B58] Perga S. Montarolo F. Martire S. Bonaldo B. Bono G. Bertolo J. (2021). Overexpression of the Ubiquitin-Editing Enzyme A20 in the Brain Lesions of Multiple Sclerosis Patients: Moving from Systemic to Central Nervous System Inflammation. Brain Pathol. 31 (2), 283–296. 10.1111/bpa.12906 33051914 PMC8018032

[B59] Polykratis A. Martens A. Eren R. O. Shirasaki Y. Yamagishi M. Yamaguchi Y. (2019). A20 Prevents Inflammasome-Dependent Arthritis by Inhibiting Macrophage Necroptosis Through its ZnF7 Ubiquitin-Binding Domain. Nat. Cel. Biol. 21 (6), 731–742. 10.1038/s41556-019-0324-3 31086261

[B60] Priem D. Devos M. Druwé S. Martens A. Slowicka K. Ting A. T. (2019). A20 Protects Cells From TNF-Induced Apoptosis Through Linear Ubiquitin-Dependent and -Independent Mechanisms. Cell Death Dis. 10 (10), 692. 10.1038/s41419-019-1937-y 31534131 PMC6751190

[B61] Priem D. van Loo G. Bertrand M. J. M. (2020). A20 and Cell Death-Driven Inflammation. Trends Immunol. 41 (5), 421–435. 10.1016/j.it.2020.03.001 32241683

[B62] Pujari R. Hunte R. Khan W. N. Shembade N. (2013). A20-Mediated Negative Regulation of Canonical NF-Κb Signaling Pathway. Immunol. Res. 57 (1-3), 166–171. 10.1007/s12026-013-8463-2 24242761

[B63] Razani B. Whang M. I. Kim F. S. Nakamura M. C. Sun X. Advincula R. (2020). Non-Catalytic Ubiquitin Binding by A20 Prevents Psoriatic Arthritis-Like Disease and Inflammation. Nat. Immunol. 21 (4), 422–433. 10.1038/s41590-020-0634-4 32205880 PMC7195210

[B64] Shembade N. Harhaj E. (2010). A20 Inhibition of NFκB and Inflammation: Targeting E2:E3 Ubiquitin Enzyme Complexes. Cell Cycle 9 (13), 2481–2482. 10.4161/cc.9.13.12269 20543575 PMC3052412

[B65] Shembade N. Harhaj E. W. (2012). Regulation of NF-Κb Signaling by the A20 Deubiquitinase. Cell. Mol. Immunol. 9 (2), 123–130. 10.1038/cmi.2011.59 22343828 PMC3532050

[B66] Shembade N. Ma A. Harhaj E. W. (2010). Inhibition of NF-kappaB Signaling by A20 Through Disruption of Ubiquitin Enzyme Complexes. Science 327 (5969), 1135–1139. 10.1126/science.1182364 20185725 PMC3025292

[B67] Shi C. S. Kehrl J. H. (2010). TRAF6 and A20 Regulate Lysine 63-Linked Ubiquitination of Beclin-1 to Control TLR4-Induced Autophagy. Sci. Signal. 3 (123), ra42. 10.1126/scisignal.2000751 20501938 PMC6335036

[B68] Slowicka K. Serramito-Gómez I. Boada-Romero E. Martens A. Sze M. Petta I. (2019). Physical and Functional Interaction Between A20 and ATG16L1-WD40 Domain in the Control of Intestinal Homeostasis. Nat. Commun. 10 (1), 1834. 10.1038/s41467-019-09667-z 31015422 PMC6478926

[B69] Sun L. Zou L. X. Han Y. C. Wu L. Chen T. Zhu D. D. (2019). A20 Overexpression Exerts Protective Effects on Podocyte Injury in Lupus Nephritis by Downregulating UCH‐L1. J. Cel. Physiol. 234, 16191–16204. 10.1002/jcp.28282 30805933

[B70] Sun S. C. (2020). A20 Restricts Inflammation via Ubiquitin Binding. Nat. Immunol. 21 (4), 362–364. 10.1038/s41590-020-0632-6 32205879

[B71] Tavares R. M. Turer E. E. Liu C. L. Advincula R. Scapini P. Rhee L. (2010). The Ubiquitin Modifying Enzyme A20 Restricts B Cell Survival and Prevents Autoimmunity. Immunity 33 (2), 181–191. 10.1016/j.immuni.2010.07.017 20705491 PMC2931361

[B72] Taylor R. C. Cullen S. P. Martin S. J. (2008). Apoptosis: Controlled Demolition at the Cellular Level. Nat. Rev. Mol. Cel. Biol. 9 (3), 231–241. 10.1038/nrm2312 18073771

[B73] Tracz M. Bialek W. (2021). Beyond K48 and K63: Non-Canonical Protein Ubiquitination. Cell. Mol. Biol. Lett. 26 (1), 1. 10.1186/s11658-020-00245-6 33402098 PMC7786512

[B74] Vande Walle L. Van Opdenbosch N. Jacques P. Fossoul A. Verheugen E. Vogel P. (2014). Negative Regulation of the NLRP3 Inflammasome by A20 Protects Against Arthritis. Nature 512 (7512), 69–73. 10.1038/nature13322 25043000 PMC4126806

[B75] Vetters J. van Helden M. J. Wahlen S. Tavernier S. J. Martens A. Fayazpour F. (2019). The Ubiquitin-Editing Enzyme A20 Controls NK Cell Homeostasis Through Regulation of mTOR Activity and TNF. J. Exp. Med. 216 (9), 2010–2023. 10.1084/jem.20182164 31296735 PMC6719426

[B76] Voet S. Mc Guire C. Hagemeyer N. Martens A. Schroeder A. Wieghofer P. (2018). A20 Critically Controls Microglia Activation and Inhibits Inflammasome-Dependent Neuroinflammation. Nat. Commun. 9 (1), 2036. 10.1038/s41467-018-04376-5 29789522 PMC5964249

[B77] Wang H H. Qi W. Zou C. Xie Z. Zhang M. Naito M. G. (2021). NEK1-Mediated Retromer Trafficking Promotes Blood-Brain Barrier Integrity by Regulating Glucose Metabolism and RIPK1 Activation. Nat. Commun. 12 (1), 4826. 10.1038/s41467-021-25157-7 34376696 PMC8355301

[B78] Wang J J. Zhong W. Su H. Xu J. Yang D. Liu X. (2021). Histone Methyltransferase Dot1L Contributes to RIPK1 Kinase‐Dependent Apoptosis in Cerebral Ischemia/Reperfusion. Jaha 10 (23), e022791. 10.1161/JAHA.121.022791 34796721 PMC9075366

[B79] Wang Y. Wen H. Fu J. Cai L. Li P. L. Zhao C. L. (2020). Hepatocyte TNF Receptor-Associated Factor 6 Aggravates Hepatic Inflammation and Fibrosis by Promoting Lysine 6-Linked Polyubiquitination of Apoptosis Signal-Regulating Kinase 1. Hepatology 71 (1), 93–111. 10.1002/hep.30822 31222801

[B80] Wang Y Y. Yang Z. Wang Q. Ren Y. Wang Q. Li Z. (2021). Bavachin Exerted Anti-Neuroinflammatory Effects by Regulation of A20 Ubiquitin-Editing Complex. Int. Immunopharmacol. 100, 108085. 10.1016/j.intimp.2021.108085 34454289

[B81] Wertz I. E. Newton K. Seshasayee D. Kusam S. Lam C. Zhang J. (2015). Phosphorylation and Linear Ubiquitin Direct A20 Inhibition of Inflammation. Nature 528 (7582), 370–375. 10.1038/nature16165 26649818

[B82] Wertz I. E. O'Rourke K. M. Zhou H. Eby M. Aravind L. Seshagiri S. (2004). De-Ubiquitination and Ubiquitin Ligase Domains of A20 Downregulate NF-KappaB Signalling. Nature 430 (7000), 694–699. 10.1038/nature02794 15258597

[B83] Wilson N. S. Dixit V. Ashkenazi A. (2009). Death Receptor Signal Transducers: Nodes of Coordination in Immune Signaling Networks. Nat. Immunol. 10 (4), 348–355. 10.1038/ni.1714 19295631

[B84] Xue Z. Xi Q. Liu H. Guo X. Zhang J. Zhang Z. (2019). miR-21 Promotes NLRP3 Inflammasome Activation to Mediate Pyroptosis and Endotoxic Shock. Cel. Death Dis. 10 (6), 461. 10.1038/s41419-019-1713-z PMC6561921 31189875

[B85] Yan K. Wu C. Ye Y. Li L. Wang X. He W. (2020). A20 Inhibits Osteoclastogenesis via TRAF6-Dependent Autophagy in Human Periodontal Ligament Cells Under Hypoxia. Cell Prolif. 53 (3), e12778. 10.1111/cpr.12778 32027437 PMC7106956

[B86] Yu X. Li W. Deng Q. Liu H. Wang X. Hu H. (2021). MYD88 L265P Elicits Mutation-Specific Ubiquitination to Drive NF-Κb Activation and Lymphomagenesis. Blood 137 (12), 1615–1627. 10.1182/blood.2020004918 33025009 PMC7995293

[B87] Yuan W. Chen Y. Zhou Y. Bao K. Yu X. Xu Y. (2021). Formononetin Attenuates Atopic Dermatitis by Upregulating A20 Expression via Activation of G Protein-Coupled Estrogen Receptor. J. Ethnopharmacol. 266, 113397. 10.1016/j.jep.2020.113397 32971159

[B88] Zhai Y. Lin P. Feng Z. Lu H. Han Q. Chen J. (2018). TNFAIP3-DEPTOR Complex Regulates Inflammasome Secretion Through Autophagy in Ankylosing Spondylitis Monocytes. Autophagy 14 (9), 1629–1643. 10.1080/15548627.2018.1458804 29940800 PMC6135570

[B89] Zhang H. Zhou X. Mcquade T. Li J. Chan F. K. Zhang J. (2011). Functional Complementation Between FADD and RIP1 in Embryos and Lymphocytes. Nature 471 (7338), 373–376. 10.1038/nature09878 21368761 PMC3072026

[B90] Zheng Z. Qu J. Q. Yi H. M. Ye X. Huang W. Xiao T. (2017). MiR-125b Regulates Proliferation and Apoptosis of Nasopharyngeal Carcinoma by Targeting A20/NF-Κb Signaling Pathway. Cel. Death Dis. 8 (6), e2855. 10.1038/cddis.2017.211 PMC5520883 28569771

[B91] Zhou J. Wu L. Y. Chen L. Guo Y. J. Sun Y. Li T. (2019). Herbs-Partitioned Moxibustion Alleviates Aberrant Intestinal Epithelial Cell Apoptosis by Upregulating A20 Expression in a Mouse Model of Crohn's Disease. World J. Gastroenterol. 25 (17), 2071–2085. 10.3748/wjg.v25.i17.2071 31114134 PMC6506586

[B92] Zhou M. He J. Shi Y. Liu X. Luo S. Cheng C. (2021). ABIN3 Negatively Regulates Necroptosis-Induced Intestinal Inflammation Through Recruiting A20 and Restricting the Ubiquitination of RIPK3 in Inflammatory Bowel Disease. J. Crohns Colitis. 15 (1), 99–114. 10.1093/ecco-jcc/jjaa131 32599618

[B93] Zou C. Mifflin L. Hu Z. Zhang T. Shan B. Wang H. (2020). Reduction of mNAT1/hNAT2 Contributes to Cerebral Endothelial Necroptosis and Aβ Accumulation in Alzheimer's Disease. Cel. Rep. 33 (10), 108447. 10.1016/j.celrep.2020.108447 33296651

